# SGLT-2 inhibitors and cardiorenal outcomes in patients with or without type 2 diabetes: a meta-analysis of 11 CVOTs

**DOI:** 10.1186/s12933-021-01430-3

**Published:** 2021-12-16

**Authors:** Dario Giugliano, Miriam Longo, Lorenzo Scappaticcio, Giuseppe Bellastella, Maria Ida Maiorino, Katherine Esposito

**Affiliations:** 1 Division of Endocrinology and Metabolic Diseases, Department of Advanced Medical and Surgical Sciences, University of Campania “Luigi Vanvitelli”, Naples, Italy; 2 PhD of Translational Medicine, Department of Advanced Medical and Surgical Sciences, University of Campania “Luigi Vanvitelli”, Naples, Italy; 3 Diabetes Unit, Department of Advanced Medical and Surgical Sciences, University of Campania “Luigi Vanvitelli”, Naples, Italy

**Keywords:** Cardiovascular outcome trials, Type 2 diabetes, SGLT-2 inhibitors, Cardiorenal outcomes

## Abstract

**Background:**

It has been suggested that sodium–glucose cotransporter 2 (SGLT-2) inhibitors reduce the cardiorenal risk in patients with type 2 diabetes (T2D). The purpose of this study is to provide an update of all large cardiovascular outcome trials (CVOTs) with SGLT-2 inhibitors to assess their cardiorenal efficacy in patients with and without T2D.

**Methods:**

An electronic search up to 30 September 2021 was conducted in PubMed, EMBASE, the Cochrane Database of Systematic Reviews, and ClinicalTrials.gov. to determine eligible trials. We included CVOTs comparing any SGLT-2 inhibitor with placebo, reporting desired cardiovascular or renal outcomes and with a follow-up duration of at least 6 months.

**Results:**

Eleven CVOTs, with data from five SGLT-2 inhibitors (empagliflozin, canagliflozin, dapagliflozin, ertugliflozin and sotagliflozin) and 77,541 participants, were included. In the overall analysis, the risk of the composite CV mortality or hospitalization for heart failure (HF) was reduced by 23% (HR = 0.77, 95% CI 0.73–0.82, P < 0.001) compared with placebo, with not significant heterogeneity (I^2^ = 26%, P = 0.20), and irrespective of the presence of T2D (P for interaction = 0.81) and age (> 65 vs ≤ 65 years, P for interaction = 0.78). The risk of CV mortality, total mortality and hospitalization for HF was significantly reduced by 16%, 13%, and 32%, respectively; similarly, the risk of the composite renal outcome was reduced by 35% (HR = 0.65, 95% CI 0.56–0.75), with moderate heterogeneity (I^2^ = 32%). In the analysis of 6 CVOTs reporting the data, the risk of major cardiovascular events (MACE) was reduced by 12%, with low heterogeneity (I^2^ = 21.2%, P = 0.19) and irrespective of the presence of established CV disease at baseline (P for interaction = 0.46).

**Conclusions:**

Therapy with SGLT-2 inhibitors in patients with cardiometabolic and renal diseases results in a sustained to moderate reduction of the composite CV death or hospitalization for HF, robust reduction of HF and renal outcomes, moderate reduction of CV mortality, total mortality and MACE.

**Supplementary Information:**

The online version contains supplementary material available at 10.1186/s12933-021-01430-3.

## Introduction

Inhibitors of the sodium–glucose co-transporter 2 (SGLT-2) were initially developed for the treatment of type 2 diabetes (T2D) for their effect of lowering blood glucose levels through increased excretion of glucose in the urine [1]. In FDA-mandated cardiovascular safety trials, also identified as cardiovascular outcome (CVOTs) trials [2], patients with T2D were typically divided by the presence or absence of established cardiovascular disease. However, once their efficacy in reducing the risk of heart failure or progression of chronic kidney disease was ascertained [3–6] and thought to be largely independent of baseline and time-dependent changes in glycated hemoglobin [7], the hypothesis emerged that their cardiorenal benefits might not necessarily be due to glucose lowering and might also be found in individuals without diabetes. The DAPA-HF (Dapagliflozin And Prevention of Adverse Outcomes in Heart Failure) trial [8] was the first one to include patients with heart failure and reduced ejection fraction irrespective of the presence of T2D: the trial showed that dapagliflozin reduced the risk of worsening heart failure events and cardiovascular death and improved symptoms in patients with or without T2D.

Since then, other CVOTs have been published which have extended the number and the typology of patients candidate to this class of drugs, giving more information to clinicians to optimize therapy and hopefully reduce their risk of cardiorenal complications. One key question is how the effects of SGLT-2 inhibitors compared in patients with and without T2D, and whether the findings of the completed trials support the hypothesis that SGLT-2 inhibition might be an effective treatment for patients with heart failure, including those without diabetes. The aim of this meta-analysis was to provide an update of all large CVOTs with SGLT-2 inhibitors to assess their cardiorenal efficacy in patients with and without T2D.

## Methods

### Search strategy and study selection

This systematic review was based on PRISMA (Preferred Reporting Items for Systematic Reviews and Meta-Analyses) guidelines [9]. The protocol has not been registered in any platform. We searched PubMed, EMBASE, the Cochrane Database of Systematic Reviews, and ClinicalTrials.gov (http://www.clinicaltrials.gov) to identify all eligible trials comparing the efficacy of SGLT-2 inhibitors with that of placebo in adult patients with or without T2D. The terms used for the research were “sodium–glucose co-transporter 2 inhibitors”, “empagliflozin”, “canagliflozin”, “dapagliflozin”, “ertugliflozin”, “sotagliflozin”, “placebo”, “cardiorenal outcomes”, “kidney outcomes”, “MACE”, “heart failure”, and “randomized controlled trials”. The search was filtered to include only randomized controlled trials (RCTs) or meta-analyses of human data. Searches were done up until September 30, 2021. We excluded observational non-randomized studies, registries, ongoing trials without results, duplicate series, meta-analysis, abstracts, and oral communications. Data were extracted by D.G. and M.L., with conflicts over study inclusion resolved by consensus. The prespecified selection criteria included: (1) randomized controlled trials comparing any SGLT-2 inhibitor with placebo; (2) RCTs reporting desired cardiovascular or renal outcomes; (3) RCTs completed before the FDA guidance of 2008 [2] and (4) follow-up duration of at least 6 months.

### Data extraction and quality assessment

Results in trial reports (primary trial results and subsequent secondary publications), and their accompanying supplementary materials, were used as the primary source of information. The retrieved data included study characteristics, characteristics of patients, interventions, and outcome measures, that included the hazard ratios (HR) and confidence intervals (CI) for cardiorenal outcomes. In more recent trials examining the effects of some SGLT-2 inhibitors (dapagliflozin or empagliflozin) independent of the presence of T2D, we did subgroups analysis assessing the effect of the SGLT-2 inhibitors on the primary outcome (CV death or hospitalization for HF) in subjects with T2D vs subjects without T2D, or in subjects > 65 years vs. subjects ≤ 65 years of age. The Cochrane Collaboration Risk-of-Bias tool was used for quality assessment of the RCTs [10], including sequence generation, allocation concealment, blinding, incomplete outcome data, and selective outcome reporting. Risk of bias was graded as unclear, high, or low.

### Data synthesis and analysis

Data were analyzed using Stata, version 16.0 (Stata Corp., College Station, TX). All statistical tests were two-sided, and P values < 0.05 were regarded as significant. The efficacy outcomes for this meta-analysis were the effect of SGLT-2 inhibitors on the incidence of the composite of CV mortality or hospitalization for HF, CV mortality, total mortality, hospitalization for HF, kidney outcomes and MACE. Subgroup analyses were done for the incidence of the composite of CV mortality or hospitalization for HF according to the presence of T2D at baseline (YES vs NO) or in subjects of 65 years of age or younger vs patients older than 65 years of age, as well as for incidence of MACE according to the presence of CV disease at baseline (YES vs NO). Hazard ratios (HRs) and 95% CI (confidence interval) for efficacy outcomes were synthesized. Heterogeneity between studies was evaluated by using the Cochran’s Q test. The proportion of variation in observed effects due to heterogeneity rather than sampling error was evaluated by using I^2^ index [11] and thresholds of I^2^ describing the degree of heterogeneity were 25% or lower (low), 26–75% (moderate), and greater than 75% (high). A Q statistic P-value of < 0.10 was considered significant Pooled summary estimates and 95% CIs for efficacy outcomes were calculated according to a random effects model using the Paule-Mandel method [12]. Publication bias was assessed with the Egger test [13]. The trim-and-fill method [14] was used to estimate the effect of publication bias (if any).

## Results

### Characteristics of the included studies

Of 160 articles screened for eligibility, 11 RCTs [8, 15–29] were eligible and included in the meta-analysis (Additional file 1: Fig. S1). Their characteristics are summarized in Table 1. The participants were all adult (> 18 years old) patients. All trials were multinational and sponsored by industry. The trials have been published between 2015 and 2021, with 3 trials published in 2021. All trials were of parallel-group, double-blind design, and their mean duration ranged from 0.75 to 4.2 years. The populations studied ranged in size from 1222 (SOLOIST-WHF) to 17,160 (DECLARE) and were of similar age (range 61.3–71.9 years). Data from 77,541 participants were included in the analysis.

**Table 1:** Characteristics of CVOTs included in the meta-analysis

	Study drug/mean follow-up (years)	Participants (n)	Age, mean or median (years)	Male sex (n, %)	Primary outcome
EMPA-REG 2015	Empagliflozin 3.1	7020	61.3	5016 71.5	MACE
CANVAS 2017	Canagliflozin 2.4	10,142	63.3	6509 64.2%	MACE
DECLARE 2019	Dapagliflozin 4.2	17,160	63.9	10,738 62.6%	MACE
CREDENCE 2019	Canagliflozin 2.6	4401	63.0	2907 66.1%	Composite renal: ESKD, doubling of serum creatinine levels, death from renal or CV causes
DAPA-HF 2019	Dapagliflozin 1.5	4744	66.0	3131 66.0%	Composite of worsening HF or CV death
DAPA CKD 2020	Dapagliflozin 2.4	4304	61.8	2879 66.9%	Composite renal: decline eGFR ≥ 50%, ESKD, death from CV or renal causes
VERTIS-CV 2020	Ertugliflozin 3.0	8246	64.4	5769 70.0%	MACE
EMPEROR-R 2020	Empagliflozin 1.5	3730	66.8	2837 76.0%	Composite of CV death and hospitalization for HF
SCORED 2021	Sotagliflozin 1.5	10,584	69.0	5896 55.7%	Composite of CV death, hospitalization for HF, urgent HT for HF
SOLOIST-WHF 2021	Sotagliflozin 0.75	1222	70.0	810 66.3%	Composite of CV death, hospitalization for HF, urgent HT for HF
EMPEROR-P 2021	Empagliflozin 2.2	5988	71.9	3317 55.4%	Composite of CV death and hospitalization for HF

MACE: major adverse cardiovascular events; ESKD: end-stage kidney disease; CV: cardiovascular; HF: heart failure; eGRF: estimated glomerular filtration rate; HT: hospitalization

EMPA-REG OUTCOME compared empagliflozin to placebo in 7020 patients with T2D and established CV disease [15, 25]. CANVAS compared canagliflozin to placebo in 10,142 patients with T2D and established CV disease or CV risk factors only [16]. DECLARE compared dapagliflozin to placebo in 17,160 patients with T2DM and established CV disease or CV risk factors only [17]. CREDENCE compared canagliflozin to placebo in 4401 patients with T2D and diabetic kidney disease [18, 26]. DAPA-HF compared dapagliflozin to placebo in 4744 patients with or without T2D and heart failure with reduced ejection fraction [8, 27, 28]. DAPA-CKD compared dapagliflozin to placebo in 4304 patients with or without T2D and chronic kidney disease [19, 29]. VERTIS-CV compared ertugliflozin to placebo in 8246 patients with T2D and established CV disease [20]. EMPEROR-R compared empagliflozin to placebo in 3730 patients with or without T2D and heart failure with reduced ejection fraction [21]. SCORED compared sotagliflozin to placebo in 10,584 patients with T2D and chronic kidney disease [22]. SOLOIST-WHF compared sotagliflozin to placebo in 1222 patients with T2D who were recently hospitalized for worsening heart failure [23]. EMPEROR-P compared empagliflozin to placebo in 5988 patients with or without T2D and heart failure with preserved ejection fraction [24]. The primary outcomes for the 11 trials are given in Table 1. According to the Cochrane Collaboration’s tool for assessing risk of bias, there was no major risk of bias in any study (Additional file 1: Fig. S2, Table S1).

### Outcomes

**Fig. 1:**
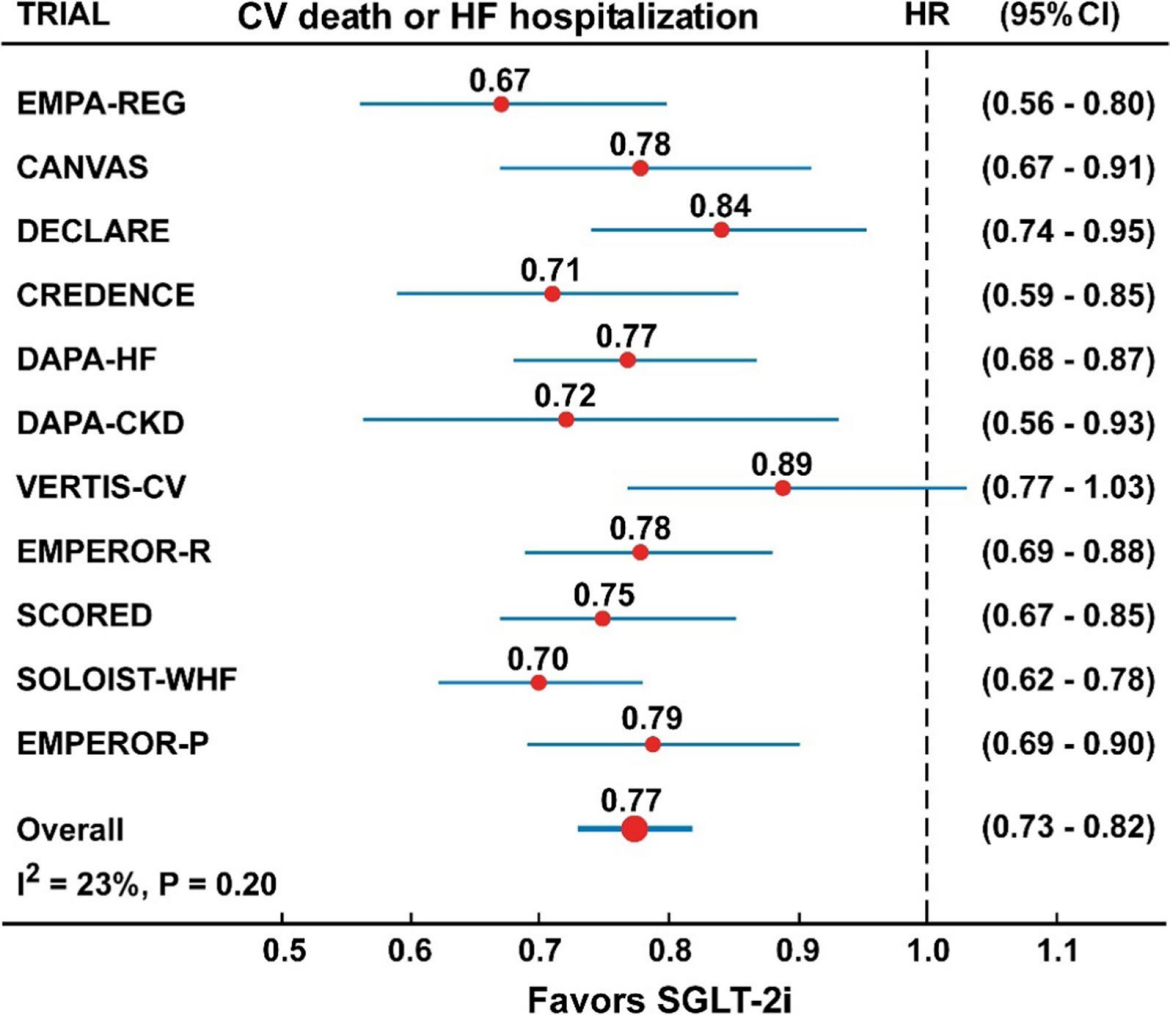
Forest plots examining the first outcome of CV death or hospitalization for heart failure in participants of 11 CVOTs irrespective of the presence of type 2 diabetes. There is a low and not significant heterogeneity in the analysis (I^2^ = 23%, P = 0.20)

In the overall analysis, including 11 trials with 77,541 participants, the risk of composite CV mortality or hospitalization for HF was reduced by 23% (HR = 0.77, 95% CI 0.73–0.82, P < 0.001) compared with placebo, with moderate and not significant heterogeneity (I^2^ = 26%, P = 0.20) (Fig. 1 and Table 2), and no evidence of publication bias (Egger test, P = 0.46). In the subanalysis of participants divided according to the presence or absence of T2D (Fig. 3 and Table 2), there was no difference in the risk of the composite CV death or hospitalization for HF between the two groups (P for interaction = 0.81). In the four trials that included participants with or without T2D (DAPA-HF, DAPA-CKD, EMPEROR-R and EMPEROR-P), treatment with dapagliflozin (DAPA) or empagliflozin (EMPEROR) was associated with 26% (HR = 0.74, 95% CI 0.64–0.84) and 23% (HR = 0.77, 95% CI 0.64–0.91) lower risk of the composite CV death or hospitalization for HF in patients with or without T2D, respectively. Similarly, in three trials (DAPA-HF, EMPEROR-R and EMPEROR-P) treatment with dapagliflozin or empagliflozin was associated with 25% (HR = 0.75, 95% CI 0.66–0.84) and 22% (HR = 0.78, 95% CI 0.68–0.88) lower risk of the composite CV death or hospitalization for HF in patients older than 65 years or 65-year-old or younger, respectively, with no significant interaction (P = 0.78) (Fig. 4 and Table 2).

**Table 2:** Results of planned meta-analyses with random effects

Outcome	Trials (n)	Estimate (HR)	95% CI	P value of HR	I^2^ (%)	P value of I^2^
CV death and HFH						
All	11	0.77	0.73–0.82	< 0.001	26.0	0.20
T2D: yes	4	0.74	0.64–0.84	< 0.001	0	0.81
T2D: no	4	0.77	0.64–0.91	< 0.001	0	0.72
Age > 65 years	3	0.75	0.66–0.84	< 0.001	0	0.92
Age ≤ 65 years	3	0.78	0.68–0.88	< 0.001	2.0	0.87
CV mortality						
All	11	0.84	0.73–0.95	0.007	42.0	0.10
Total mortality						
All	11	0.87	0.74–0.98	0.009	45.0	0.07
HF hospitalization						
All	10	0.68	0.62–0.74	< 0.001	0	0.98
Kidney outcomes						
All	10	0.65	0.56–0.75	< 0.001	35.0	0.10
MACE						
All	6	0.88	0.83–0.93	< 0.010	21.2	0.19
Prior CVD	5	0.87	0.82–0.92	0.001	12.0	0.35
No prior CVD	3	0.93	0.83–1.07	0.326	55.1	0.10

CV, cardiovascular; HFH, hospitalization for heart failure; HR, hazard ratio; CI, confidence intervals; T2D, type 2 diabetes; MACE, major cardiovascular events

In the overall analysis including all the 11 CVOTs, the risk of CV mortality (Fig. 5 and Table 2), was reduced by 16% (HR = 0.84, 95% CI 0.73–0.95) by treatment with SGLT-2 inhibitors, with moderate and significant heterogeneity (I^2^ = 0.42%, P = 0.07) and some evidence of publication bias (Egger test, P = 0.047). The trim-and-fill method indicated that this publication bias did not change the statistical significance of the estimate (HR 0.85, 95% CI 0.74–0.96). Similarly, the risk of total mortality (Fig. 6 and Table 2), was reduced by 13% (HR = 0.87, 95% CI 0.74–0.98) by treatment with SGLT-2 inhibitors, with moderate heterogeneity (I^2^ = 0.45%) and some evidence of publication bias (Egger test, P = 0.042). The trim-and-fill method indicated that this publication bias did not change the statistical significance of the estimate (HR 0.88, 95% CI 0.75–0.97).

**Fig. 2:**
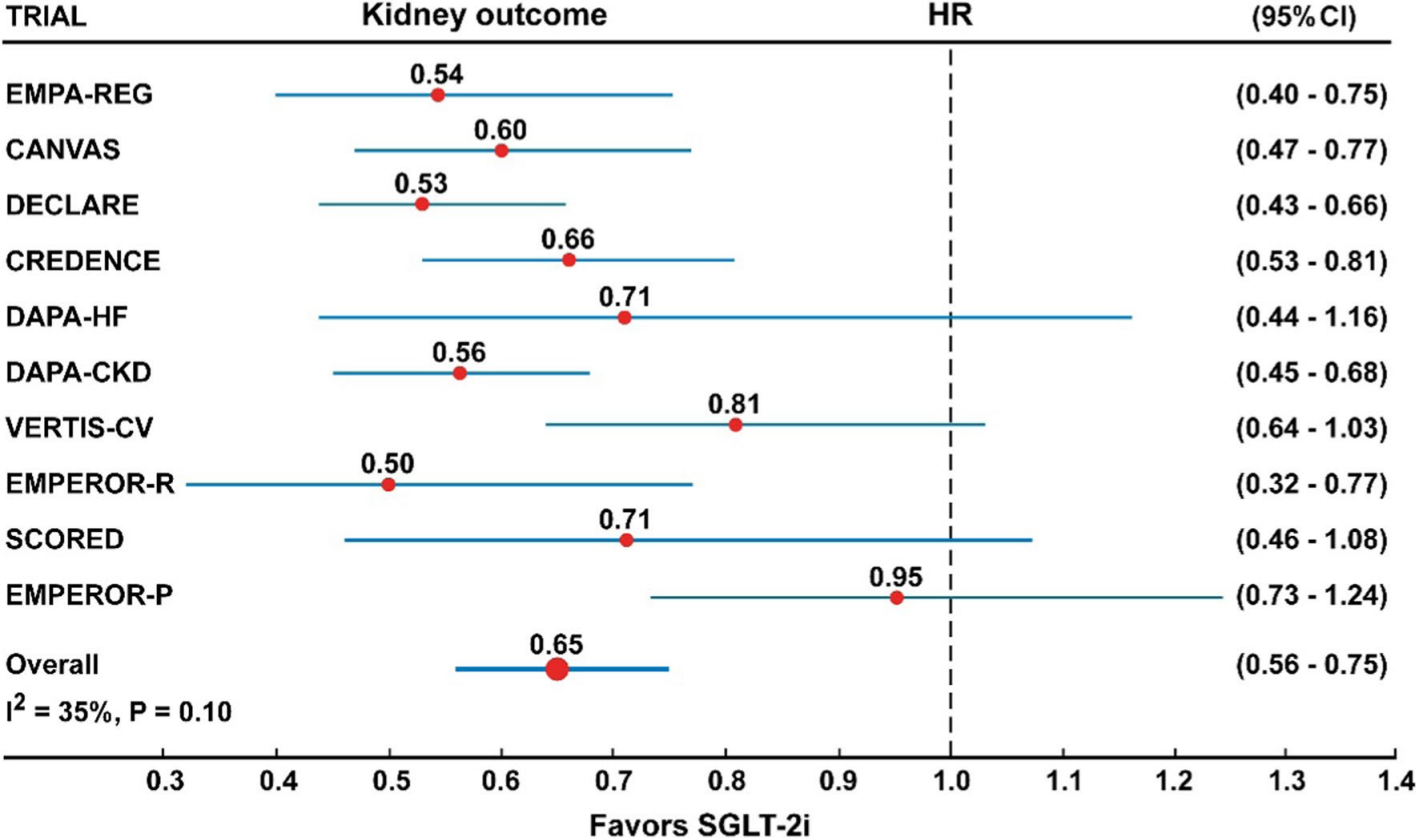
Forest plots examining the kidney outcome in participants of ten CVOTs irrespective of the presence of type 2 diabetes and diabetic kidney disease. There is a moderate and borderline significant heterogeneity in the analysis (I^2^ = 35%, P = 0.10)

In the analysis of 10 trials with SGLT-2 inhibitors (Fig. 7, Table 2), the risk of hospitalization for HF was reduced by 32% (HR = 0.68, 95% CI 0.62–0.74), with no heterogeneity (I^2^ = 0%) and no evidence of publication bias (Egger test, P = 0.85). Similarly, the risk of the composite renal outcome (Fig. 2, Table 2) was reduced by 35% (HR = 0.65, 95% CI 0.56–0.75), with moderate but not significant heterogeneity (I^2^ = 35%) but no evidence of publication bias (Egger test, P = 0.15).

In the analysis of 6 CVOTs (EMPA-REG, CANVAS, DECLARE, CREDENE, VERTIS-CV, SCORED) (Table 2), the risk of MACE was reduced by 12%, with low heterogeneity (I^2^ = 21.2%, P = 0.19). There was no difference in the risk of MACE according to the presence or absence of established CV disease at baseline (P for interaction = 0.46).

**Fig. 3:**
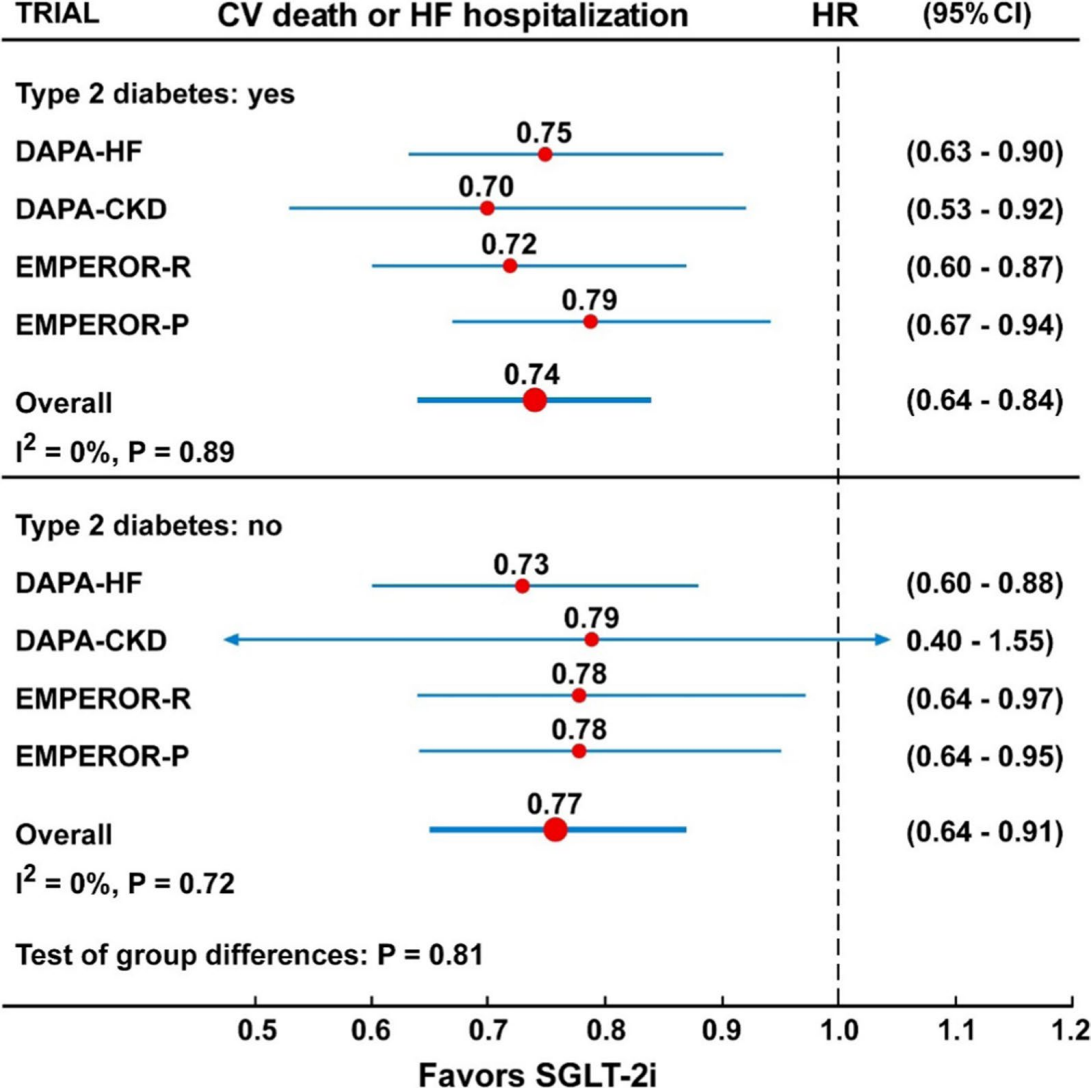
Forest plots examining the composite outcome of cardiovascular death or hospitalization for heart failure in participants of four CVOTs with (top) or without (bottom) type 2 diabetes. There is no heterogeneity in the analyses (I^2^ = 0%) and difference between the two groups (P interaction = 0.81)

**Fig. 4:**
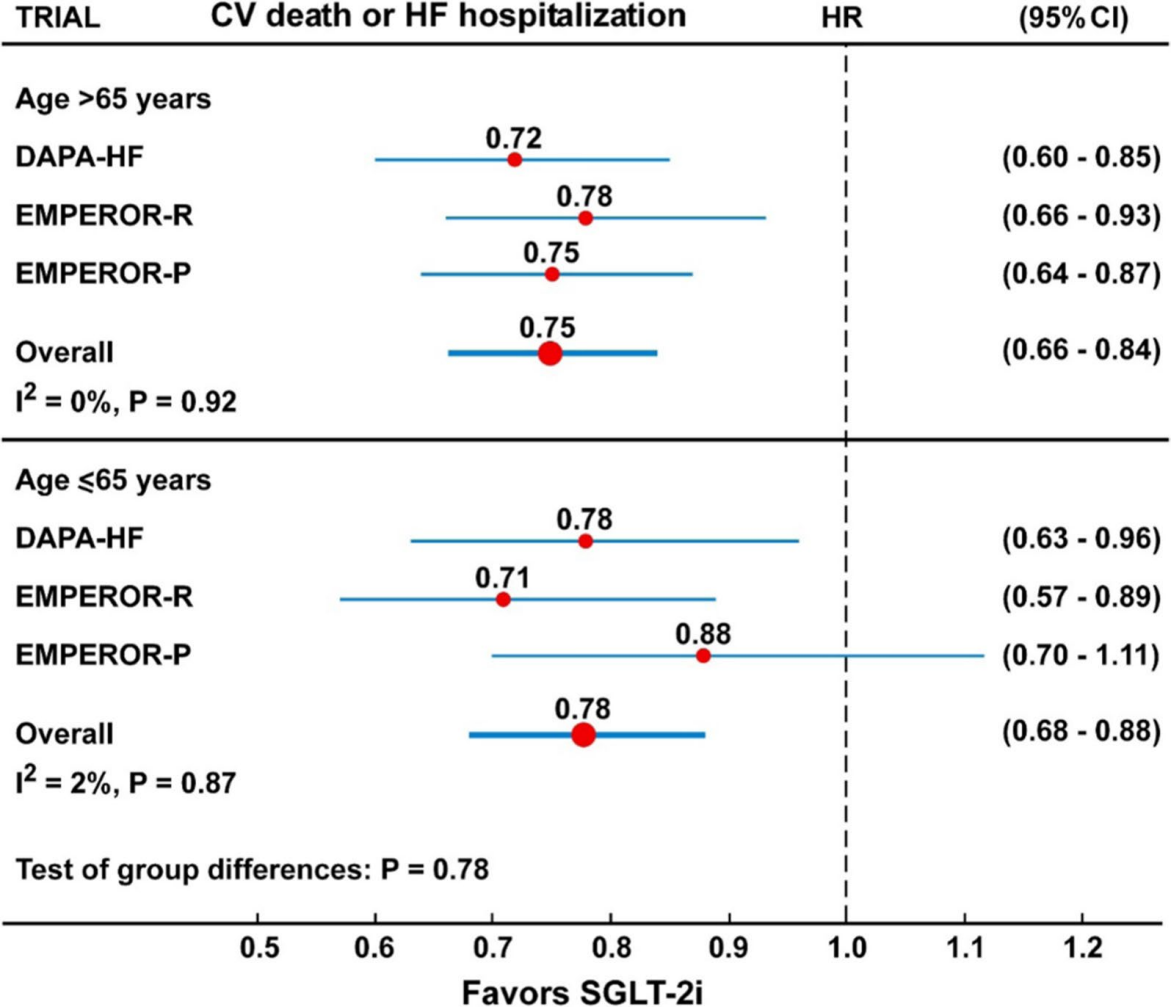
Forest plots examining the composite outcome of cardiovascular death or hospitalization for heart failure in participants of three CVOTs with age > 65 years (top) or ≤ 65 years (bottom). There is no heterogeneity in the analyses (I^2^ = 0–2%) and difference between the two groups (P interaction = 0.78)

**Fig. 5:**
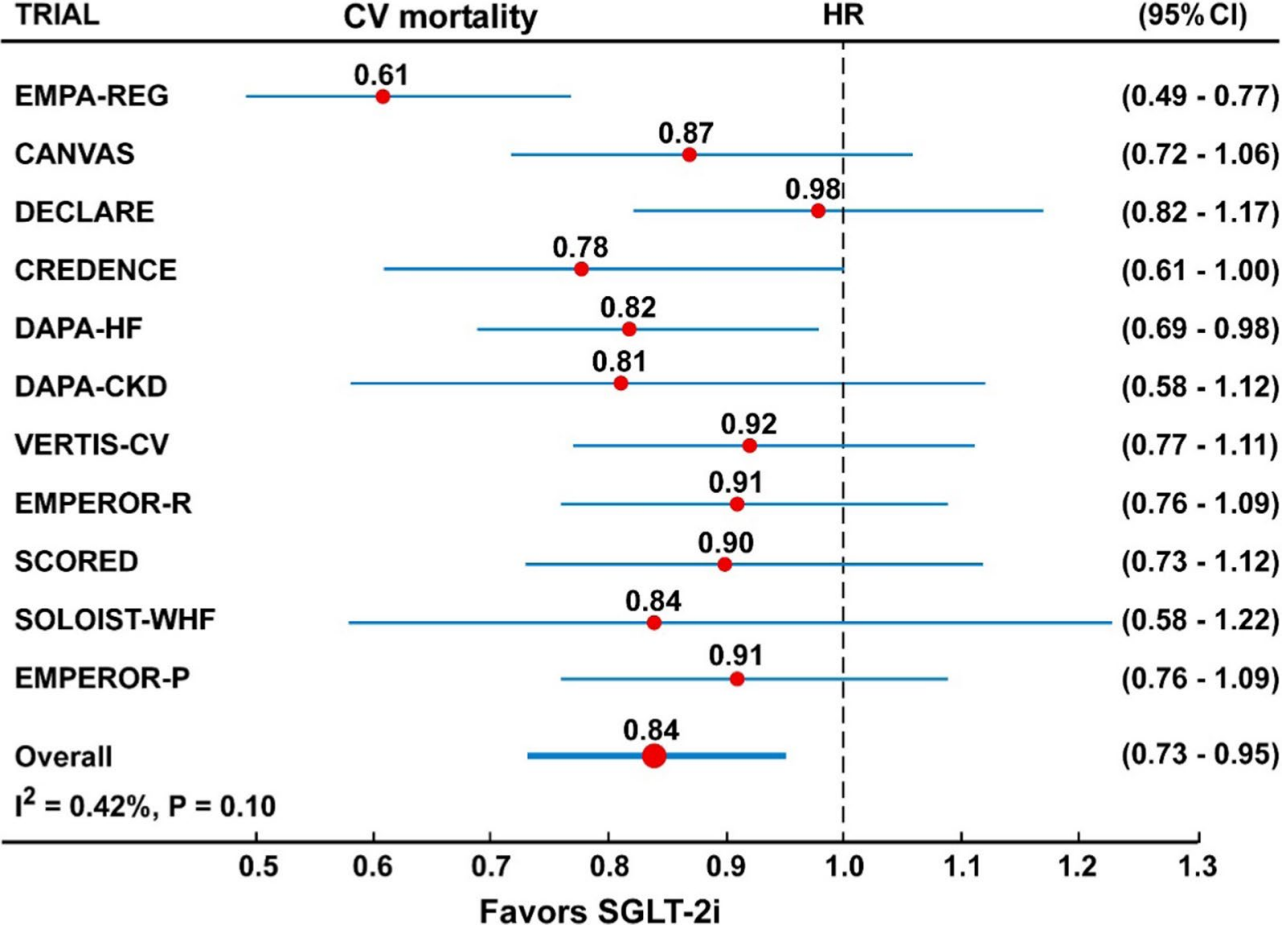
Forest plots examining the outcome cardiovascular mortality in participants of 11 CVOTs. There is moderate heterogeneity in the analyses (I^2^ = 42% of borderline significance)

**Fig. 6:**
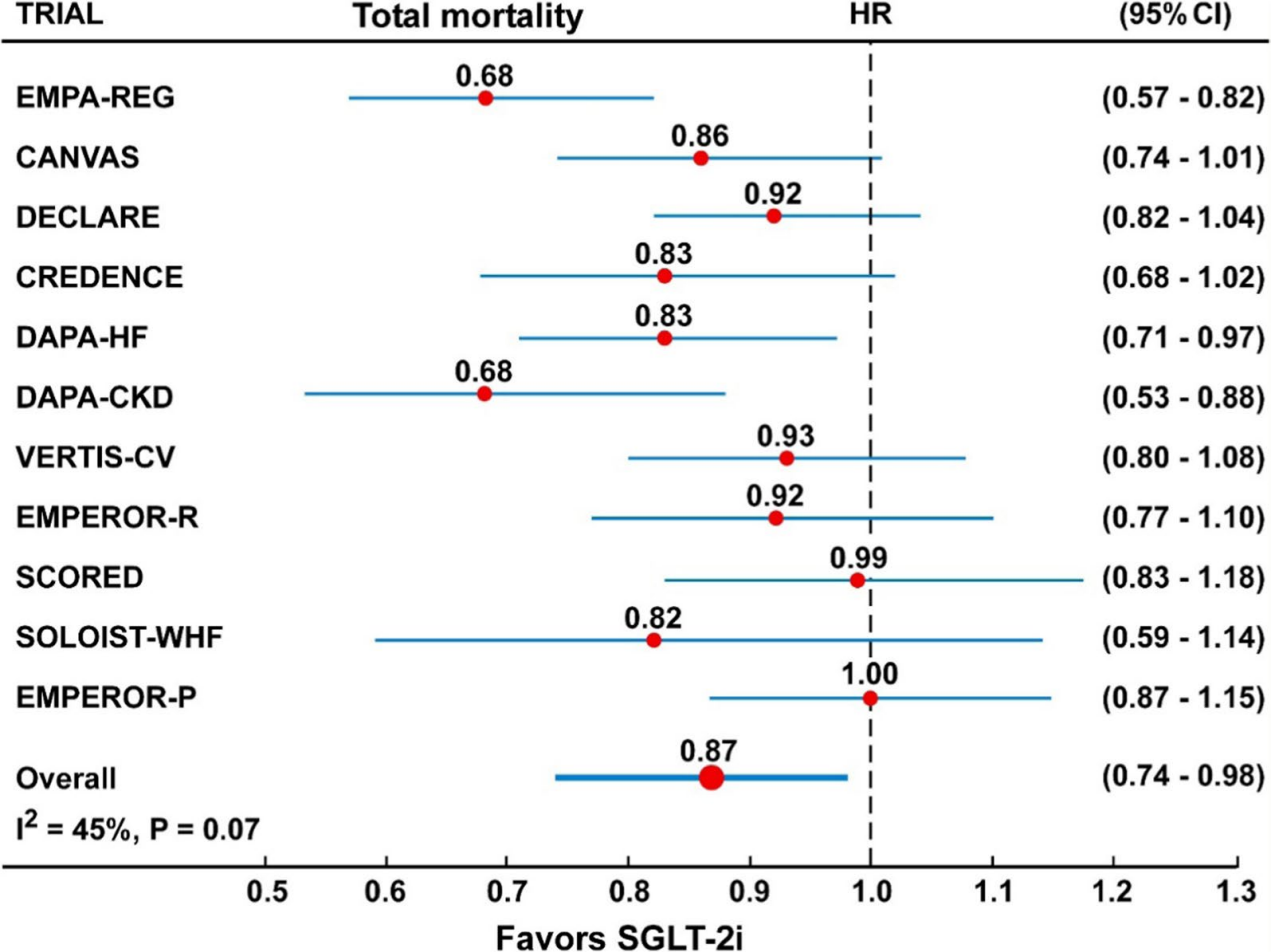
Forest plots examining the outcome cardiovascular mortality in participants of 11 CVOTs. There is moderate and significant heterogeneity in the analyses (I^2^ = 45%)

**Fig. 7:**
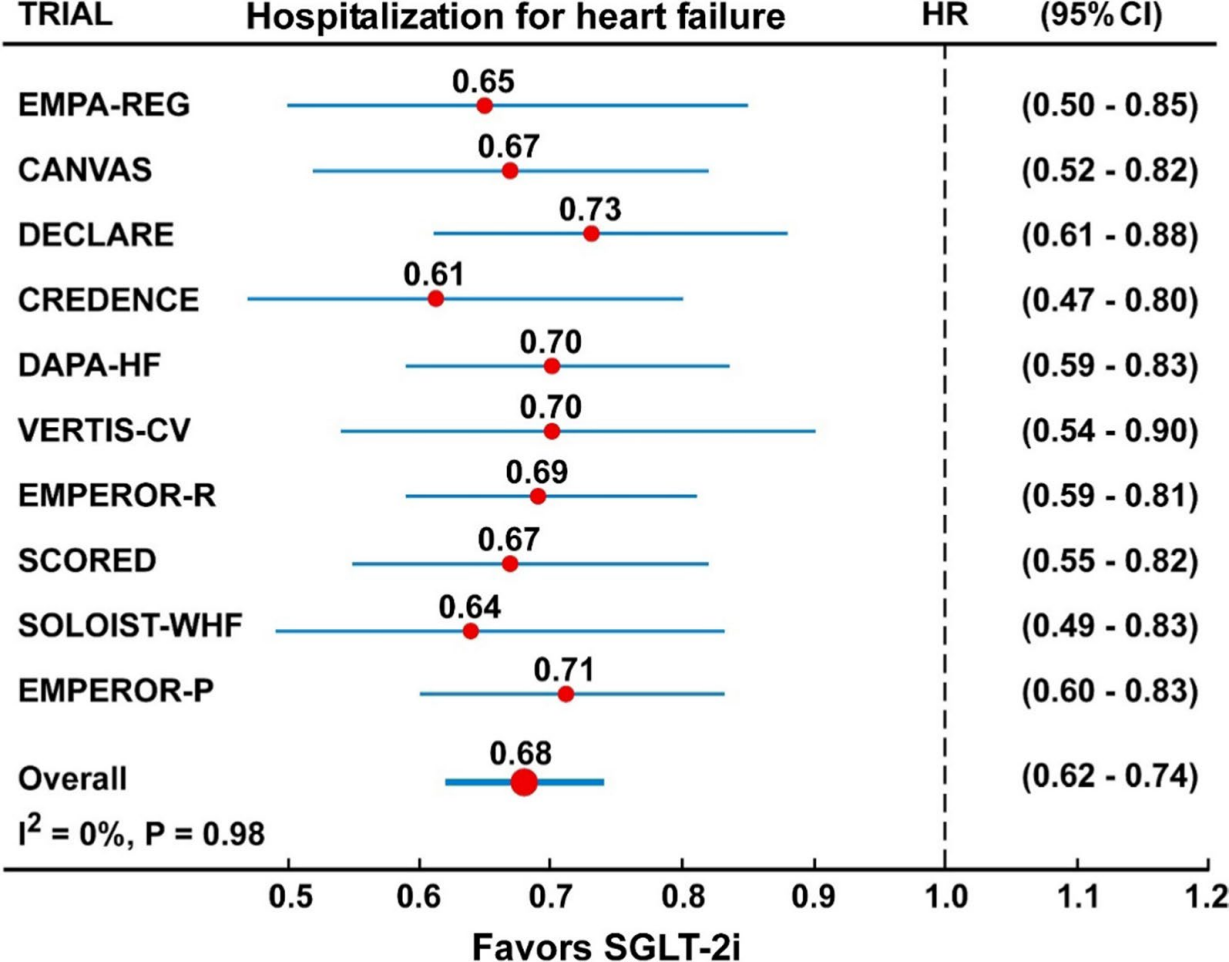
Forest plots examining the outcome hospitalization for heart failure in participants of ten CVOTs. There is a null heterogeneity in the analyses (I^2^ = 0%)

In all analyses shown in Figs. 1, 2, 3, 4, 5, 6, 7, the Hazard ratios for the specific outcomes of two sotagliflozin trials (SCORED and SOLOIST-HF), when included, were like to those of the other SGLT-2 inhibitors (empagliflozin, canagliflozin, dapagliflozin, ertugliflozin).

## Discussion

The present meta-analysis includes the most recent published large RCTs (SCORED, SOLOIST-WHF and EMPEROR-P), thus providing the most contemporary assessment of the total available evidence for SGLT-2 inhibitor therapy and cardiorenal outcomes in patients with or without T2D. The findings of 11 CVOTs involving 77,541 patients show that treatment with SGLT-2 inhibitors reduced the risk of the composite CV death or hospitalization for HF by 23% in the overall population, with low and not significant heterogeneity, suggesting a plausible class effect for the outcome. This interpretation seems also supported by the subgroup analyses indicating the lack of significant difference in the reduced risk of the composite outcome in patients with or without T2D, or in subjects of 65 years of age or younger vs those older than 65 years of age. Although participants in the EMPEROR-P trial [24] were grouped by different age-threshold (> 70 years vs ≤ 70 years), this may not have altered the estimation of the Hazard Ratio given the absence of heterogeneity and interaction between groups.

To date, the most meaningful class effect of SGLT-2 inhibitors appears to be that on HF hospitalization for the following reasons: (1) the reduced risk for HF hospitalization is > 25% in every CVOT published until now (range 27–39%); (2) there is a complete absence of heterogeneity (I^2^ = 0%, P = 0.98) in the meta-analysis; (3) the confidence intervals of the HR are very close to the point estimate; and (4) the reduced risk of hospitalization for HF is significant in every CVOTs. So, we can be confident that the beneficial effect of SGLT-2 inhibitors to reduce the risk of hospitalization for HF is a class effect and is independent of the diabetes status, heart status (presence or absence of HF or established CV disease at baseline) and kidney status (presence or absence of chronic kidney disease at baseline) [5, 30, 31].

A recent meta-analysis [32] of 8 CVOTs pooled data of 65,587 patients and found that treatment with SGLT-2 inhibitors reduced the risk of the renal composite endpoint by 39%, including worsening renal function, end-stage kidney disease and death from CV or renal causes, adding evidence to their nephroprotective effect. While confirming nephroprotection by SGLT-2 inhibitors with a pooled reduced risk of renal outcome by 35%, the present meta-analysis including ten CVOTs reporting the renal outcome seems to question the class effect of SGLT-2 inhibitors for the following reasons: (1) the reduced risk of renal outcome is not significant in every CVOTs; (2) apart from canagliflozin, all other inhibitors (dapagliflozin, ertugliflozin, sotagliflozin and empagliflozin) fail to produce a significant nephroprotection in at least one CVOT; (3) there is moderate and borderline significant heterogeneity (I^2^ = 35%, P = 0.10) in the present meta-analysis, probably as a consequence of the different criteria used to define the kidney outcome; and (4) the worst results, in terms of wideness of confidence intervals, are observed in patients with HF, independent of the presence of T2D. In particular, the beneficial effect of empagliflozin on the renal outcome was significantly greater in the EMPEROR-R than in the EMPEROR-P trial [33], suggesting a major protective effect of the drug in patients with HF and a reduced ejection fraction. It is possible that the definition of the renal outcome may have played some role in the striking discordance between the effect of empagliflozin on heart failure and renal outcomes in EMPEROR-P trial. This discordance is extraordinarily puzzling as the effects of SGLT-2 inhibitors on hospitalization for HF and renal outcome had consistently tracked together in previous CVOTs. As empagliflozin reduced the risk of HF hospitalization in these two populations of patients with HF (reduced or preserved ejection fraction) irrespective of their baseline diabetic status, it is conceivable that nephroprotection is not the principal mechanism by which empagliflozin may prevent HF hospitalization [34]. It has already been shown that SGLT-2 inhibitors led to greater benefits in patients with NYHA class II than in patients with NYHA class III or IV, and that the reduced HF composite outcome is independent of LVEF level (< 40%, 40% to < 50%, or ≥ 50%) [35, 36].

Several hypotheses have been formulated in the attempt to explain the cardiorenal protective effects of SGLT-2 inhibitors, including, although not limited to a diuretic effect [37], altered substrate utilization and cellular signaling though increased lipolysis in adipose tissue with subsequent generation of ketone bodies [38], increased erythropoietin, hemoglobin, and hematocrit levels which can improve tissue oxygenation [39] and improved the lipid profile and decreasing uric acid level [40]. Moreover, the increase in the delivery of sodium to the macula densa results in vasoconstriction of the afferent arteriolar with a subsequent reduction in the intraglomerular pressure [41]. Irrespective of the exact mechanism, the improvement in cardiorenal outcomes by SGLT-2 inhibitors in patients with and without T2D suggest inherent protective properties. To date, it is not yet possible to clearly identify subpopulations of patients without T2D that would benefit the most from treatment with SGLT-2 inhibitors.

This study has potential limitations that include the use of aggregate trial-data levels and some difference in the exact inclusion/exclusion criteria and definition of outcomes among trials. Moreover, not all CVOTs have published the subgroup data for all outcomes and therefore some trials are not included in the analysis for individual endpoints. Strengths of the present meta-analysis are the inclusion of all CVOTs published by September 30, 2021, the very large number of participants, the high quality of the trials which minimizes the risk of bias, and the absence of significant heterogeneity in most analyses, which ranged from absent to low or moderate.

The clinical relevance of these results seems also highlighted by the evidence that for some outcomes the clinical benefit is consistent irrespective of the presence of T2D, advanced age, and cardiorenal disease. On the basis of results of CVOTs, the FDA has approved dapagliflozin (May 5, 2020) and empagliflozin (August 18, 2021) to reduce risk for CV death and HF hospitalization in adults with HF with reduced ejection fraction regardless of whether they have diabetes [42, 43].

## Conclusions

Therapy with SGLT-2 inhibitors results in a sustained to moderate reduction of the composite CV death or hospitalization for HF, robust reduction of HF and renal outcome, and moderate reduction of CV, total mortality and MACE. The shift from a common antihyperglycemic drug to an agent with the likely indication of cardiorenal protection is under way and close to the goal. However, it is unlikely that future studies will significantly change the present scenario based on 11 CVOTs with more than 75,000 patients.

## Supplementary Information

**Additional file 1.** Supplemental file.

## Data Availability

All data generated or analyzed during this study are included in this published article and in its Additional file.

## Abbreviations

CVOTs: Cardiovascular outcome trials
SGLT-2: Sodium–glucose cotransporter-2
T2D: Type 2 diabetes
MACE: Major cardiovascular events
HR: Hazard ratios
HF: Heart failure
