# Supplementary material for: SGLT-2 inhibitors and cardiorenal outcomes in patients with or without type 2 diabetes: a meta-analysis of 11 CVOTs
Source: Cardiovasc Diabetol. 2021 Dec 16;20:236. doi: 10.1186/s12933-021-01430-3 (PMC8680308; doi:10.1186/s12933-021-01430-3)
Supplement: Supplementary file 1 — Additional file 1. Supplemental file. [file 12933_2021_1430_MOESM1_ESM.docx]

**SGLT-2 inhibitors and cardiorenal outcomes in patients with or without type 2 diabetes: a meta-analysis of eleven CVOTs**

Dario Giugliano, Miriam Longo, Lorenzo Scappaticcio, Giuseppe Bellastella, Maria Ida Maiorino, Katherine Esposito

**Additional Material**

*Additional Figure S1……………………………………………………………………………………………. pag. 2*

*Additional Figure S2……………………………………………………………………………………………… pag. 3*

*Additional Table S1……………………………………………………………………………………………… pag. 4*

*PRISMA Statement…………………………………………………………………………………………………….. pag. 5*

*Protocol…………………………………………………………………………………………………………………….. pag. 8*

**Screening**

**Included**

**Eligibility**

**Identification**

Titles/abstracts assessed for

eligibility

(n = 160)

Excluded (n = 135)

- Reviews, comments, editorials (30)
- No CVOT or not contributory secondary analysis (56)
- Design manuscript (24)
- Meta-analyses (20)

Full-text article assessed for eligibility

(n = 25)

Records identified through database search (n = 160)

Full-text articles excluded (n=14)

- Further analysis of the main trial (7)
- Not reporting cardiorenal outcomes (7)

Trials included (n = 11)

**Key Search Terms:**

“SGLT-2 inhibitors” OR “empagliflozin” OR “canagliflozin” OR “dapagliflozin” OR “ertugliflozin” OR “sotagliflozin” OR “type 2 diabetes” OR “CVOTs” OR “MACE” OR “renal outcome” OR “cardiovascular outcome” OR “heart failure” OR “Trial”

**Figure S1.** Process of studies’ selection


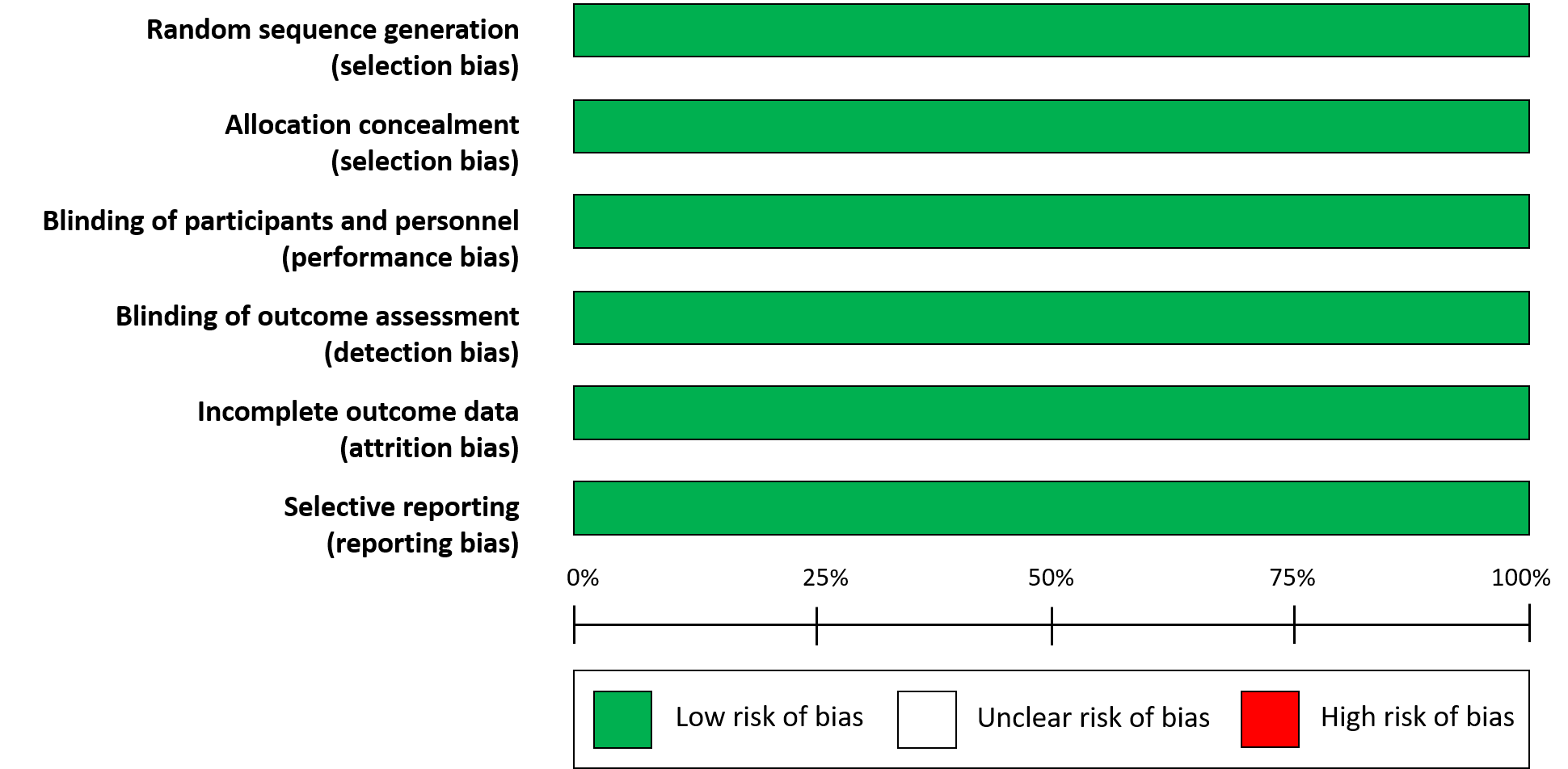


**Figure S2.** Cochrane risk of bias (graph) for the 11 trials

| **Table S1.** Summary of risk of bias assessment | | | | |  |  |  |
| --- | --- | --- | --- | --- | --- | --- | --- |
| Study ID | Random sequence generation* | | Allocation concealment* | Blinding of participants and personnel° | Blinding of outcome assessment° | Incomplete outcome data° | Selective reporting° |
| EMPA-REG, 2015 | L | | L | L | L | L | L |
| CANVAS, 2017 | L | | L | L | L | L | L |
| DECLARE, 2019 | L | | L | L | L | L | L |
| CREDENCE, 2019 | L | | L | L | L | L | L |
| DAPA-HF, 2019 | L | | L | L | L | L | L |
| DAPA-CKD, 2020 | L | | L | L | L | L | L |
| VERTIS-CV, 2020 | L | | L | L | L | L | L |
| EMPEROR-R, 2020 | L | | L | L | L | L | L |
| SCORED, 2021 | L | | L | L | L | L | L |
| SOLOIST-WHF, 2021 | L | | L | L | L | L | L |
| EMPEROR-P, 2021 | L | | L | L | L | L | L |
| L= low risk of bias; U= unclear risk of bias; H= high risk of bias | | | | |  |  |  |
| *Risk of bias assessment for random sequence generation and allocation concealment is | | | | | | |  |
| performed at the study level. | | |  |  |  |  |  |
| °Risk of bias assessment for blinding of participants and personnel, blinding of outcome | | | | | | |  |
| assessment, incomplete outcome data, and selective reporting are for the primary outcome. | | | | | | |  |
|  | |  |  |  |  |  |  |

**PRISMA checklist**

| **Section/topic** | | | **#** | **Checklist item** | **Reported on page #** |  |
| --- | --- | --- | --- | --- | --- | --- |
| **TITLE** | | | | |  |  |
| Title | | | 1 | Identify the report as a systematic review, meta-analysis, or both. | 1 |  |
| **ABSTRACT** | | | | |  |  |
| Structured summary | | | 2 | Provide a structured summary including, as applicable: background; objectives; data sources; study eligibility criteria, participants, and interventions; study appraisal and synthesis methods; results; limitations; conclusions and implications of key findings; systematic review registration number. | 2 |  |
| **INTRODUCTION** | | | | |  |  |
| Rationale | | | 3 | Describe the rationale for the review in the context of what is already known. | 4 |  |
| Objectives | | | 4 | Provide an explicit statement of questions being addressed with reference to participants, interventions, comparisons, outcomes, and study design (PICOS). | 4 |  |
| **METHODS** | | | | |  |  |
| Protocol and registration | 5 | | | Indicate if a review protocol exists, if and where it can be accessed (e.g., Web address), and, if available, provide registration information including registration number. | 5 |  |
| Eligibility criteria | 6 | | | Specify study characteristics (e.g., PICOS, length of follow-up) and report characteristics (e.g., years considered, language, publication status) used as criteria for eligibility, giving rationale. | 5 |  |
| Information sources | 7 | | | Describe all information sources (e.g., databases with dates of coverage, contact with study authors to identify additional studies) in the search and date last searched. | 5 |  |
| Search | 8 | | | Present full electronic search strategy for at least one database, including any limits used, such that it could be repeated. | 5 |  |
| Study selection | 9 | | | State the process for selecting studies (i.e., screening, eligibility, included in systematic review, and, if applicable, included in the meta-analysis). | 5 |  |
| Data collection process | 10 | | | Describe method of data extraction from reports (e.g., piloted forms, independently, in duplicate) and any processes for obtaining and confirming data from investigators. | 5-6 |  |
| Data items | 11 | | | List and define all variables for which data were sought (e.g., PICOS, funding sources) and any assumptions and simplifications made. | 5-6 |  |
| Risk of bias in individual studies | 12 | | | Describe methods used for assessing risk of bias of individual studies (including specification of whether this was done at the study or outcome level), and how this information is to be used in any data synthesis. | 6 |  |
| Summary measures | 13 | | | State the principal summary measures (e.g., risk ratio, difference in means). | 6 |  |
| Synthesis of results | 14 | | | Describe the methods of handling data and combining results of studies, if done, including measures of consistency (e.g., I^2^) for each meta-analysis. | 6 |  |
| Risk of bias across studies | | 15 | | | Specify any assessment of risk of bias that may affect the cumulative evidence (e.g., publication bias, selective reporting within studies). | 6 |
| Additional analyses | | 16 | | | Describe methods of additional analyses (e.g., sensitivity or subgroup analyses, meta-regression), if done, indicating which were pre-specified. | 6 |
| **RESULTS** | | | | | |  |
| Study selection | | 17 | | | Give numbers of studies screened, assessed for eligibility, and included in the review, with reasons for exclusions at each stage, ideally with a flow diagram. | 7, Figure S1 |
| Study characteristics | | 18 | | | For each study, present characteristics for which data were extracted (e.g., study size, PICOS, follow-up period) and provide the citations. | 7; (Table 1) |
| Risk of bias within studies | | 19 | | | Present data on risk of bias of each study and, if available, any outcome level assessment (see item 12). | (Figure S2, Table S1) |
| Results of individual studies | | 20 | | | For all outcomes considered (benefits or harms), present, for each study: (a) simple summary data for each intervention group (b) effect estimates and confidence intervals, ideally with a forest plot. | 8 |
| Synthesis of results | | 21 | | | Present results of each meta-analysis done, including confidence intervals and measures of consistency. | 8 |
| Risk of bias across studies | | 22 | | | Present results of any assessment of risk of bias across studies (see Item 15). | 7-8 |
| Additional analysis | | 23 | | | Give results of additional analyses, if done (e.g., sensitivity or subgroup analyses, meta-regression [see Item 16]). | 8-9 |
| **DISCUSSION** | | | | | |  |
| Summary of evidence | | | 24 | | Summarize the main findings including the strength of evidence for each main outcome; consider their relevance to key groups (e.g., healthcare providers, users, and policy makers). | 9-12 |
| Limitations | | | 25 | | Discuss limitations at study and outcome level (e.g., risk of bias), and at review-level (e.g., incomplete retrieval of identified research, reporting bias). | 9-12 |
| Conclusions | | | 26 | | Provide a general interpretation of the results in the context of other evidence, and implications for future research. | 9-12 |
| **FUNDING** | | | | | |  |
| Funding | | | 27 | | Describe sources of funding for the systematic review and other support (e.g., supply of data); role of funders for the systematic review. | 12 |

**Protocol for the systematic literature search about the effect of CVOTs with SGLT-2 inhibitors on cardiorenal outcomes in patients with type 2 diabetes.**

- Broad question 1: what is the effect of SGLT-2 inhibitors, as compared with placebo, on cardiorenal risk in patients with or without type 2 diabetes?
- Broad question 2: what is the effect of SGLT-2 inhibitors, as compared with placebo, on mortality?
- Specific question 1: what is the effect of SGLT-2i, as compared with placebo, on the composite of CV death or hospitalization for heart failure, irrespective of the presence of type 2 diabetes and age? What is the of SGLT-2i on hospitalization for heart failure, renal outcome and MACE?
- Specific question 2: what is the effect of SGLT-2i, as compared with placebo, on cardiovascular of total mortality in the whole population?

The answer to these points was sought by evaluating cardiovascular outcome trials (CVOTs) that compared SGLT-2i with placebo in people with or without type 2 diabetes. Risk of composite CV death or hospitalization for heart failure (hazard ratio and 95% confidence interval), total and CV mortality, hospitalization foe HF, composite renal outcome (ESKD, doubling of serum creatinine levels, death from renal or cardiovascular causes) and MACE (major CV events) were the endpoints of the comparison.

*The review followed the outlines of PICO (study characteristics):*

1. Population: the population to be included in the review consisted of subjects with type 2 diabetes at baseline.
2. Exposure: SGLT-2i compared with placebo
3. Comparisons: subjects with type 2 diabetes.
4. Outcomes: Risk of MACE and composite renal endpoint

Published articles were considered eligible for this review if they were: randomized controlled trials comparing an SGLT-2 inhibitor (empagliflozin, dapagliflozin, canagliflozin, ertugliflozin, sotagliflozin) with placebo; 2) RCTs reporting desired cardiovascular or renal outcomes; 3) RCTs completed before the FDA guidance of 2008 and 4) follow-up duration of at least 6 months.
